# Editorial for the Special Issue on Network on Chip (NoC) and Reconfigurable Systems

**DOI:** 10.3390/mi14091780

**Published:** 2023-09-17

**Authors:** Abdelkrim Zitouni

**Affiliations:** Department of Physics, College of Science & Humanities, Imam Abdulrahman Bin Faisal University, P.O. Box 1982, Jubail 31961, Saudi Arabia; azitouni@iau.edu.sa

In a multiprocessor System-on-Chip (SoC), efficient communication between the associated processors has to be addressed at all levels of the system design to guarantee global interconnection. Recently, massive attention has been given to the network-on-chip (NoC) as the communication architecture of SoC because it is scalable compared to the communication bus. These new paradigms extensively minimize SoC power consumption and end-to-end delay. Particularly in 3D NoC-based applications, system modules come from multiple clock domains. These modules communicate in different layers while achieving real-time traffic. This trend can be well guaranteed by the Globally Asynchronous Locally Synchronous (GALS) paradigm. Process manufacturers also prefer NoC since they present communication bottlenecks under process variation.

Nevertheless, in NoC architectures, concurrent tasks are scheduled and mapped to a set of selected IP addresses. Partial and Dynamic Reconfiguration (DPR), supported by several FPGAs, allows reconfiguring some parts of the FPGA at runtime. This allows for the optimization of the system area by reusing the same hardware NoC resources for different application tasks. Performance, resource usage, and power consumption must be considered by the designer. This may be well simplified by developing a NoC platform while meeting all the constraints of a NoC reconfigurable system. However, this implies an increased system design complexity that can be seen at different design levels, such as designing reconfigurable application tasks, reconfigurable architecture regions, reconfiguration control, etc. Therefore, there is a need for a Model-Driven Engineering (MDE) approach that automates and simplifies the designer’s work while meeting all the constraints of a reconfigurable design.

In this Special Issue on Network on Chip (NoC) and Reconfigurable Systems, we include eight papers covering different aspects related to 3D NoC [1,2], GALS NoC [3], NoC routing [4,5,6], application of NoC to the Internet of Things (IoT) [7], and dynamic partially reconfigurable architectures [8].

In [1], Fang et al. present a 3D NoC partitioning and a double particle swarm optimization (DPSO) algorithm, which can delineate the 3D NoC mapped region and integrate the characteristics of neighborhood search and genetic algorithms in order to solve the problem of a particle swarm algorithm falling into local optimal solutions. This work aims to automate the mapping of applications into 3D NoC topologies, which constitutes an important new direction for research in the field of 3D NoC. In [2], Savva et al. propose a novel 3D NoC router that combines buffered and bufferless routing with approximate priority comparison when deflecting flits. The proposed algorithm achieves additional performance and area gains due to the reduced logic required for approximate priority comparison in flit deflections. Relatively to traditional bufferless routers, this router is characterized by a higher clock frequency and a reduced area.

Li et al. describe a multistage packet reordering (MPR) approach, which reduces the transmission latency and hotspots caused by local reordering. This work contributes to solving the packet reordering problem, which represents one of the keys to ensuring the quality of service (QoS) for GALS NoC. This work improved thermal efficiency with reduced hardware resource consumption [3].

In [4], Zhou et al. present a PCCNoC (Packet Connected Circuit NoC), a low-latency and low-overhead NoC based on packet and circuit switching. This algorithm offers flexible routing and zero overhead of data transmission latency, making it suitable for high-end heterogeneous multi-core real-time SoC at various system scales. The aim is to solve the problems of high-end heterogeneous multi-core real-time NoC and to meet the need for low latency, high throughput, and flexibility. Compared with typically available packet-switched NoC, PCCoC sees 242% improved performance and 97% latency reduction while keeping the silicon cost relatively low. Romanov et al. [5] present a new high-level model, Newxim, for exploring NoCs with any topology. Two methods for solving the problem of cyclic dependencies in circulant topologies, which limit their applications for NoCs due to the increased possibility of deadlocks, are proposed. The first method of dealing with deadlocks is universal and applicable to any topology; it is based on bypassing blocked sections of the network on an acyclic subnetwork. The second method, Ring-Split, considers the features of circulant topologies. This work considers the usage of circulant topologies as a promising deadlock-free topology for networks-on-chip (NoCs). In [6], Effiong et al. design an R-NoC (NoC Router) inspired by real-life multi-lane traffic roundabouts and consists of lanes shared by multiple input/output ports to maximize buffering resource utilization. This router is implemented on 45 nm CMOS technology. This work aims to improve network performance. In fact, buffers consume significant power/area and are often underutilized, especially in cases of applications with non-uniform traffic patterns, thus leading to performance degradation for such applications.

Hou presents in [7] the use of the Lorenz chaotic system to generate chaotic signals and apply them to the encryption of the communication of the Internet of Things (IoT) terminal sensor nodes configured in a NoC architecture. A sophisticated proportional–integral–derivative (PID) controller and a quasi-sliding mode controller (QSMC) have been designed to synchronize the master-slave chaotic systems for decrypting the signals. Finally, in [8], Dalbouchi et al. study the reconfigurable feature of FPGAs (Field-Programmable Gate Arrays) that made them a desirable solution for implementing adaptive SoC by proposing a model-driven design flow that guides the designer through the description of the different elements of a reconfigurable system. The proposed model is based on high-level modeling using an extended version of the MARTE (Modeling and Analysis of Real-Time and Embedded Systems) UML (Unified Modeling Language) profile. The flow allows centralized and decentralized reconfiguration decision-making solutions, allowing it to adapt various reconfigurable system constraints. This design flow was validated through a reconfigurable video watermarking application. The automatically generated watermarking system allowed a good trade-off between resource usage, power consumption, execution time, and image quality compared to static implementations.

We hope that this Special Issue on Network on Chip (NoC) and Reconfigurable Systems will offer readers a good outline of the actual state of the art in this fast-increasing area of research and an introduction to some of the latest techniques developed in the domain.
